# Cell Membrane Adaptations Mediate β-Lactam-Induced Resensitization of Daptomycin-Resistant (DAP-R) *Staphylococcus aureus* In Vitro

**DOI:** 10.3390/microorganisms9051028

**Published:** 2021-05-11

**Authors:** Nagendra N. Mishra, Arnold S. Bayer, Sarah L. Baines, Ashleigh S. Hayes, Benjamin P. Howden, Christian K. Lapitan, Cassandra Lew, Warren E. Rose

**Affiliations:** 1 Division of Infectious Diseases, The Lundquist Institute at Harbor-UCLA Medical Center, Torrance, CA 90502, USA; abayer@lundquist.org (A.S.B.); christian.lapitan@lundquist.org (C.K.L.); 2 David Geffen School of Medicine, University of California (UCLA), Los Angeles, CA 90024, USA; 3 Doherty Applied Microbial Genomics, Department of Microbiology and Immunology, The University of Melbourne at the Peter Doherty Institute for Infection and Immunity, Melbourne, VIC 3004, Australia; bainess@unimelb.edu.au (S.L.B.); ashleigh.hayes@unimelb.edu.au (A.S.H.); bhowden@unimelb.edu.au (B.P.H.); 4 School of Pharmacy, University of Wisconsin-Madison, Madison, WI 53705, USA; clew@wisc.edu (C.L.); warren.rose@wisc.edu (W.E.R.)

**Keywords:** CM lipids, daptomycin resistance, resensitization, MRSA

## Abstract

The reversal of daptomycin resistance in MRSA to a daptomycin-susceptible phenotype following prolonged passage in selected β-lactams occurs coincident with the accumulation of multiple point mutations in the *mprF* gene. MprF regulates surface charge by modulating the content and translocation of the positively charged cell membrane phospholipid, lysyl-phosphatidylglycerol (LPG). The precise cell membrane adaptations accompanying such β-lactam-induced *mprF* perturbations are unknown. This study examined key cell membrane metrics relevant to antimicrobial resistance among three daptomycin-resistant MRSA clinical strains, which became daptomycin-susceptible following prolonged exposure to cloxacillin (‘daptomycin-resensitized’). The causal role of such secondary *mprF* mutations in mediating daptomycin resensitization was confirmed through allelic exchange strategies. The daptomycin-resensitized strains derived either post-cloxacillin passage or via allelic exchange (vs. their respective daptomycin-resistant strains) showed the following cell membrane changes: (i) enhanced BODIPY-DAP binding; (ii) significant reductions in LPG content, accompanied by significant increases in phosphatidylglycerol content (*p* < 0.05); (iii) no significant changes in positive cell surface charge; (iv) decreased cell membrane fluidity (*p* < 0.05); (v) enhanced carotenoid content (*p* < 0.05); and (vi) lower branched chain fatty acid profiles (antiso- vs. iso-), resulting in increases in saturated fatty acid composition (*p* < 0.05). Overall, the cell membrane characteristics of the daptomycin-resensitized strains resembled those of parental daptomycin-susceptible strains. Daptomycin resensitization with selected β-lactams results in both definable genetic changes (i.e., *mprF* mutations) and a number of key cell membrane phenotype modifications, which likely facilitate daptomycin activity.

## 1. Introduction

*S. aureus* is a leading cause of bacteremia and other endovascular infections including endocarditis, vascular catheter sepsis, and intracardiac device infections [1,2,3]. Of note, methicillin-resistant *S. aureus* (MRSA) comprise up to one-half of these cases [4]. Complications associated with the standard-of-care anti-MRSA antibiotic, vancomycin (e.g., poor overall clinical responses, persistent bacteremia, renal toxicity), has resulted in increased use of alternative MRSA therapies such as daptomycin (DAP), which are accompanied by excess health-care expenditures [5,6,7].

Daptomycin is an effective treatment against invasive *S. aureus* infections, including MRSA [8,9]. It remains the only rapidly bactericidal anti-MRSA antibiotic with consistent efficacy in bacteremia and infective endocarditis [7,10]. Due to the increasing usage of DAP, a number of reports describing DAP-resistant (R) MRSA strains emerging during DAP therapy, associated with treatment failures, have been cited, particularly in cases of osteomyelitis and endocarditis [11,12,13,14]. Since DAP has been considered a last resort antibiotic treatment option for severe MRSA infections, the evolution of DAP-R can be very problematic for patients. In addition, newer anti-MRSA antibiotics either have no proven efficacy in severe bacteremic syndromes (telavancin, tedizolid), have evoked documented clinical and microbiologic resistance (ceftaroline), and/or have issues regarding optimal dosing regimens in systemic MRSA infections (dalbavancin, oritavancin) [15,16,17,18,19]. Therefore, it is important to design strategies that can both salvage the bactericidal activity of DAP as well as prevent the development of DAP-R during treatment. One novel finding that has frequently accompanied the evolution of DAP-R in *S. aureus* is the so-called “see-saw” effect [20]; in this phenomenon, increasing DAP MICs are associated with a concomitant and significant enhancement in β-lactam susceptibility, despite retention of the *mecA* gene encoding for PBP2a-mediated β-lactam resistance [20,21,22,23]. This latter finding underscores both the likely complex adaptations that underlie the DAP-R phenotype and the possibility of modifying the DAP-R to a DAP-susceptible (S) phenotype pharmacologically [24].

Previous reports demonstrate that the DAP-R phenotype in MRSA features a number of mutations in global regulatory genes, as well as in genes involved in CM and/or cell wall homeostasis [25,26,27]. The most frequently cited mutations in MRSA associated with DAP-R are perturbations in conserved “hot spots” within the multipeptide resistance factor (*mprF*) gene [25,26,27]. MprF is responsible for lysinylating the anionic phospholipid, phosphatidylglycerol (PG), into the cationic lysyl-phosphatidylglycerol (LPG) species, by increasing LPG synthesis and/or its translocation into the outer CM leaflet [28,29,30,31,32,33,34,35,36]. This results in an enhancement of net positive surface charge and putative formation of a more charge-repulsive milieu against calcium-DAP oligomeric aggregates, which reduces DAP insertion into the target CM [28,29,30,31,32,33,34,35,36,37]. Of note, DAP-R MRSA strains often undergo several other phenotypic modifications in CM metrics including in CM order (fluidity/rigidity), carotenoid content, and fatty acid composition [32,33,34,35,36].

β-lactam antibiotics enhance the activity of DAP in vitro and in vivo against both DAP-S and DAP-R MRSA [38]. The mechanisms associated with this combinatorial interaction are incompletely understood but have been suggested to include (i) β-lactam-induced enhancement of DAP binding to the target bacterial surface [38] or (ii) more targeted binding to those CM regions where DAP is most effective (i.e., the cell divisome) [39,40,41]. In this regard, we have recently reported a novel genetic linkage related to β-lactam resensitization of DAP-R strains [41,42,43]. In those investigations, the penicillin-binding protein (PBP)-1-specific β-lactam, cloxacillin (LOX), was uniquely effective in restoring a DAP-S phenotype [41]; the genetic perturbations occurring in parental DAP-R strains (with single *mprF* mutations), when exposed to prolonged LOX passage, featured an accumulation of additional mutations in *mprF* and/or mutations in the divisome gene, *div1b* [41]. Of interest, previous in vitro studies determined that dual *mprF* mutations in DAP-R strains reversed this phenotype to DAP-S [29].

The current study examined a selected cadre of relevant CM phenotypic modifications that occurred during the transition from the DAP-R-to-DAP-S phenotype, which is induced by prolonged passage in vitro to LOX. 

## 2. Materials and Methods

**Bacterial strains and growth conditions.** The bacterial strains used in this investigation are listed in Table 1. Three isogenic DAP-S parental (WT)/DAP-R MRSA strain pairs (J01/J03, D592/D712, and C24/C25) were utilized in this study, representing: (i) clinically derived DAP-S isolates and their respective DAP-R variants emerging during DAP therapy and (ii) the most common clonal complex (CC) types causing clinical infections in the United States (USA100 and USA300, CC5 and CC8, respectively) [29]. In addition, for each strain-pair, we included a DAP-S variant selected by prolonged LOX passage (see below). Comparing each DAP-S parental strain with its DAP-R mutant revealed a single mutation within the *mprF* locus in this latter strain. In contrast, the post-LOX-passage DAP-S variant had accumulated an additional *mprF* mutation. These detailed genotypic characteristics are further described elsewhere [41]. 

For most experiments in this investigation, Mueller–Hinton broth (MHB; Difco, Rock Island, IL, USA) was utilized [41]. However, for studies quantifying surface charge, as well as CM carotenoid and fatty acid contents and phospholipids, an enriched media was required (brain heart infusion broth (BHI; Bacto, Mount Pritchard, NSW, Australia)), with all cultures being aerated at 37 °C. Carotenoid and fatty acid are major determinants of CM biophysical properties (e.g., CM rigidity/fluidity); in turn, these characteristics impact key events such as susceptibility to antimicrobials (e.g., DAP and β-lactams), staphylococcal pathogenesis, and organism responses to environmental stressors [34,35,36]. 

**In vitro passaging of DAP-R strains in LOX.** As described before [41], DAP-R isolates, J03, D712, and C25 were passaged in LOX for 28 days, with the post-passage strains now exhibiting DAP-S by MIC testing. The concentrations of LOX used for serial passage (1.4 mg/L) represented sub-MIC free average levels achieved in human serum (fC_avg_) for each DAP-R isolate. Cultures were grown overnight, diluted, and resuspended in fresh media (MHB supplemented with 25 mg/L calcium, 12.5 mg/L magnesium and 2% NaCl) to a total volume of one ml for daily passage as previously described [41]. All experimental passage experiments were performed in triplicate. The previously published mutations acquired during prolonged LOX passage, as well as DAP and LOX susceptibilities of the strain-sets are described in Table 1 [41].

**Construction of *mprF*-mutants by allelic exchange.** Introduction of a secondary *mprF* mutation into the three DAP-R backgrounds (which contain a single *mprF* mutation) was conducted using the allelic exchange protocol developed by Monk and Stinear [44] with modification (full detail provided in supplementary methods). Oligonucleotides tailed with sequence complementary to pIMAY-Z were designed to amplify a ≈ 1.2 kb region surrounding the secondary *mprF* mutation (Table S1) [45], in which the LOX passaged DAP-resensitized strains served as a donor for the sequence. *E. coli* strain IM08B was used for electrocompetent transformation [46].

Suspected *mprF* double mutant (DM) colonies and cultures of the parent DAP-R strains used for allelic exchange underwent whole genome sequencing (WGS) to confirm their genotype (NextSeq; Illumina, San Diego, CA, USA), as previously described [41]. Both the primary (from the DAP-R parent) and secondary (introduced) *mprF* mutations were confirmed in all three backgrounds. Only one off-target missense mutation was identified: a A308V amino acid change in predicted gene FFX42_RS09315 in the D712 *mprF* DM (reference D592, CC5 background).

**Surface charge.** The relative positive cell surface charge of the three DAP-S/DAP-R/LOX-DAP-resensitized strain-sets was assayed using the standard polycationic cytochrome C (Cyt C) binding assay as described elsewhere [32,33,34]. Briefly, *S. aureus* strains were grown in BHI broth to stationary phase, washed with MOPS (3-morpholinopropane-1-sulfonic acid) buffer (pH 7.0), resuspended in the same buffer at OD578 ≈ 1.0, and then incubated with 0.5 mg/mL of Cyt C for 10 min. Then, the residual quantity of Cyt C remaining in the bacterial supernatant was measured spectrophotometrically at OD530 nm, as described previously [32,33,34]. A decrease in the quantity of Cyt C binding (i.e., more cation in the supernate) reflects a greater positively charged bacterial surface [32,33,34]. The data are presented as mean (±SD) of bound Cyt C. A minimum of three independent experiments was performed on separate days.

**CM phospholipid (PL) composition and amino-PL (LPG) asymmetry.** The lipid extraction methodology has been described before [32,33,34,35,36]. *S. aureus’* major PLs (lysyl-phosphatidylglycerol (LPG); phosphatidylglycerol (PG); and cardiolipin (CL)) were separated using two-dimensional thin-layer chromatography (2D-TLC), using a unique solvent system as previously described [32,33,34,35,36]. The isolated PL spots on TLC plates were scraped, digested at 180 °C for 3 h with 0.3 mL 70% perchloric acid to convert into the inorganic form of phosphate, and quantified spectrophotometrically at OD_660_ by phosphate estimation assay. The identification of all spots on the TLC plate were carried out by exposure to iodine vapors and by spraying with CuSO_4_ (100 mg/mL) containing 8% phosphoric acid (*v*/*v*) and heated at 180 °C.

Fluorescamine labeling (a CM-impermeant UV fluorophore that binds to amino PLs, such as LPG, in the outer CM leaflet and is a measure of LPG translocation) was performed, using the same 2D TLC plates [32,33,34,35,36]. The percentage of fluorescamine-labeled LPG was calculated from the phosphorus data relative to total PLs. In general, LPG resides predominantly in the inner leaflet of the *S. aureus* CM; however, variable amounts of LPG can be translocated from the inner-to-outer CM leaflet to maintain lipid homeostasis. Fluorescamine labeling of outer CM (O)-LPG was detected by using a UV detector (365 nm). Fluorescamine-labeled LPG alters its mobility characteristics, and its ability to be detected by ninhydrin staining is attenuated. Unlabeled LPG (inner CM [I]-LPG), was visualized by ninhydrin staining. The identity of each of the major TLC spots was made in relation to known positive control PLs. Data were presented as the mean (±SD) percentages of the three major PLs (Total LPG + PG + CL = 100%).

**BODIPY-DAP fluorescence microscopy.** DAP binding was performed using confocal microscopy with BODIPY-labeled DAP. Cells were incubated with BODIPY-labeled DAP as previously described [37]. The cells were concentrated 20-fold, and 3 μL was placed on a glass slide. Slides were set with prolonged diamond antifade mountant and a #1.5 glass coverslip. Images were collected using a Leica SP8 3X STED Super-Resolution Confocal Microscope using a 489 nm laser line and 510–579 nm emission with 660 nm depletion. ImageJ was utilized to measure integrated fluorescence density of 30 cells, and corrected cell total fluorescence was calculated.

**Quantification of carotenoids:** The extraction and quantification of CM carotenoids was performed as described previously [33,34,35,36]. Strains were grown at the late stationary phase in BHI broth at 37 °C for 18–24 h and then harvested, washed, and pelleted in PBS by centrifugation. The post-removal of extra liquid from final pellets occurred by inversion for 2 min; then, pellet wet-weights were recorded. After 0.5 mL methanol was added to 0.1 g of the bacterial pellet, cells were vortexed vigorously and centrifuged. The upper layer of methanol extract was collected for the quantification of overall carotenoid content [33,34,35,36], which was determined spectrophotometrically at OD_450_ [33,34,35,36]. These assays were performed a minimum of five times for individual strains on different days.

**Fatty acid content.***S. aureus* cells were saponified, methylated, and fatty acid esters were extracted into hexane as detailed [43]. The resulting methyl ester mixtures were separated by gas chromatography [43]. Fatty acids were identified by a well-characterized microbial identification system. The external calibration standards (a mixture of the straight-chained saturated fatty acids from 9 to 20 carbons in length and five hydroxy acids) and individual known fatty acids were used to calibrate equivalent chain length (ECL) data for fatty acid identification [43]. The ECL value for each fatty acid are represented as a function of its elution time in reference to the elution time of known standards of straight-chain fatty acids [43]. Short, medium, and long-chain saturated fatty acids were grouped per carbon number [43]. ECLx = (Rtx − Rtn/Rt (n + 1) − Rtn) + n, where Rtx is the retention time of x, Rtn is the retention time of the saturated fatty acid methyl ester preceding x, and Rt(n + 1) is the retention time of the saturated fatty acid methyl ester eluting after x. FA data represent the means (±SD) from a minimum of two independent determinations on different days. Data were expressed as the percentage of the major FAs (branch chain [BC] FA + saturated [S]FA + unsaturated [U]FA = 100%). FAs present representing <1% of the total content were not included in the data analysis.

**CM order (fluidity/rigidity).** CM order profoundly impacts the interactions of DAP with the *S. aureus* CM [33,34,35,36]. MRSA strain-sets were grown overnight in BHI broth at 37 °C, harvested by centrifugation, and then washed with PBS. A whole-cell suspension of the MRSA strains was prepared at an OD_600_ = 1.0 (≈10^8^ CFU mL^−1^). CM fluidity was measured by polarizing spectrofluorometry utilizing the fluorescent probe, 1,6-diphenyl-1,3,5-hexatriene (DPH) (excitation and emission wavelengths of 360 and 426 nm). The detailed methods for quantifying DPH incorporation into CMs and the calculations of the degree of fluorescence polarization (polarization index (PI)) are described elsewhere [21,22]. An inverse relationship occurs between PI values and CM fluidity (i.e., lower PI equates to a greater extent of CM fluidity) [32,33,34,35,36]. These experiments were carried out a minimum of four times for each strain-set on different days.

**Statistical Analyses.** The two-tailed Student’s t-test was used for statistical analysis of all quantitative data. *p* values of ≤0.05 were considered “significant”.

## 3. Results

**Figure 1:**
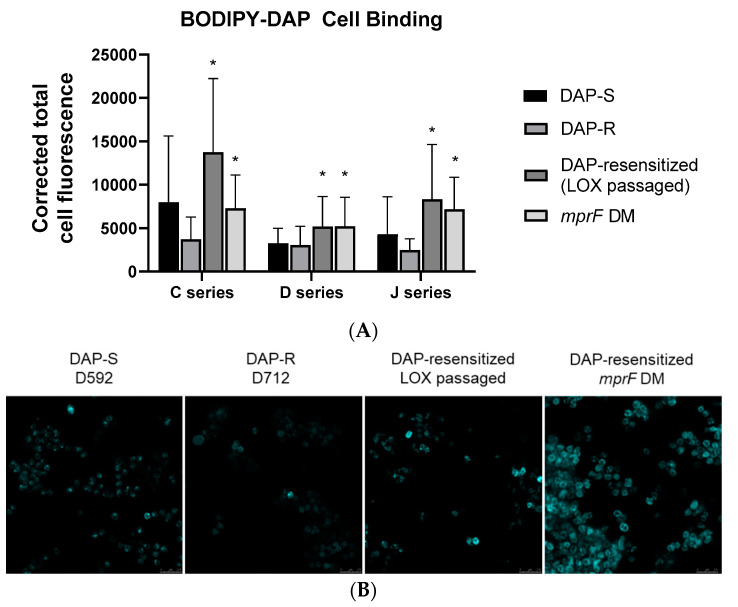
Binding of BODIPY-labeled daptomycin (BODIPY-DAP) to *S. aureus* study strains. (**A**) Corrected total cell fluoresence of BODIPY-DAP binding. (**B**) Representative confocal microscopy images of BODPY-DAP binding in the well-characterized D series strains. * *p* < 0.01 vs. DAP R-strain.

**Resensitization of DAP-R MRSA to DAP-S.** As previously published, LOX resensitized all DAP-R strains (J03, D712, and C25) to DAP (DAP-resensitized) after serial passage for 28 days in vitro (Table 1) [41]. The secondary *mprF* mutations derived from LOX passage strains were reintroduced (*mprF* DM) into the respective DAP-R parental strains. This allelic replacement resulted in a reduction of DAP MICs to levels similar to the post-LOX passage strains. This enhanced DAP resensitization correlated with increased BODIPY-DAP binding by confocal microscopy (Figure 1).

**CM phospholipid (PL) content.** As expected, the DAP-R variants showed CM PL profiles featuring increased total LPG content vs. the respective DAP-S parental strainconsistent with other previously described DAP-R strains (Table 2); this reflects the gain-in-function impacts typical of single *mprF* mutations [42]. Of interest, the increased total LPG content in this mutant was associated with enhanced synthesis but not increased outer CM translocation (data not shown). In contrast, the DAP-resensitized variants, either derived post-LOX passage or via allelic replacement, demonstrated reductions in overall synthesis of LPG to levels compatible with the DAP-S parental strain (Table 2). This CM PL profile in the DAP-resensitized strains is consistent with the documented accumulation of an additional *mprF* mutation in these strains [41], resulting in a decrease-of-function phenotype.

**Cell surface charge.** As shown in Table 3, in two of the three DAP-R strains, more unbound cytochrome C (reflecting a more positive cell surface charge) was observed vs. their respective DAP-S parental strains. In strains with secondary *mprF* mutation derived post-LOX passage as well as in the allelic reintroduction strains (*mprF* DM), a more negative surface charge was observed as compared to respective DAP-R strains (*p* < 0.05) and similar to the DAP-S parental strains.

**Fatty acid content.** We observed considerable shifts in the patterns of saturated fatty acids (SFAs) as well as iso- and anteiso-branched chain fatty acid (BCFA) profiles among the DAP-resensitized strains in comparison to both the parental DAP-S and their respective DAP-R mutant strains (Table 4 and Table S2). Interestingly, the DAP-resensitized strains had elevated SFA content as compared to their respective DAP-R mutants. However, changes in SFA content in DAP-resensitized strains vs. respective DAP-S parental strains were minimal. A significant decrease in the proportion of total anteiso-BCFAs was noted in the DAP-resensitized strains versus the corresponding DAP-R mutants (*p* < 0.01). The DAP-resensitized strains and their DAP-S parental strains had similar levels of anteiso-BCFAs. In addition, no consistent pattern of iso-BCFAs differences was observed among the strain-sets (Table 4 and Table S2). It should be noted that increased anteiso-SFA, reduced iso-SFA, and SFA content correlates with more fluid CM in *S. aureus* [34,35,36].

**CM carotenoids.***S. aureus* utilizes its carotenoid content to modulate its CM order (i.e., the more carotenoid content, the more rigid the CM) to resist the microbicidal action of DAP and other cationic peptides [33,34,35]. The DAP-R strains had lower carotenoid content vs. their corresponding DAP-S strains (Table 5), paralleling our prior data [36]. This is also consistent with the more fluid CMs noted below in the DAP-R vs. their respective DAP-S parental strains. There was a significant enhancement in CM carotenoid content among the DAP-resensitized variants, either derived from LOX passage or allelic exchange vs. their respective DAP-R strains (Table 5) (*p* < 0.05). These data in line with the re-establishing of a more rigid, parental-level CM.

**CM order (fluidity/rigidity).** There appears to be an optimal degree of CM order for the interaction of most CM-targeting cationic peptides, including calcium-complexed DAP [34,35,36]. As seen with other DAP-R mutants of MRSA [47], the three DAP-R mutants in our study displayed more fluid CMs vs. each respective parental DAP-S strain (Table 5). The DAP-resensitized strains overall had a shift in CM order toward a less fluid (more rigid) CM, similar to the pattern of their respective DAP-S parental strains (Table 5).

## 4. Discussion

Our recent studies suggest that β-lactams can synergize with DAP against DAP-R MRSA through the inhibition of PBP-1 without necessarily enhancing DAP binding [38]. Furthermore, distinct β-lactam antibiotics (such as LOX) can uniquely resensitize DAP-R MRSA strains to a DAP-S phenotype [41], which is a phenomenon that was only previously found through in vitro genetic manipulation of *mprF* [28,29,30]. Of note, WGS confirmed that DAP-resensitized strains frequently acquire additional *mprF* mutations in either their translocase or synthase domains [41]. The accumulation of such secondary *mprF* mutations in our LOX-post passage variants correlated with a significant drop in DAP MICs, which is often below that of the DAP-S parental strains [41]. The emergence of DAP-R in MRSA and mutations in *mprF* are often associated with distinct compensatory changes in PL composition, surface charge, and CM biophysics (e.g., fluidity profiles). Understanding the CM-associated adaptations in DAP-R mutants when exposed to selected β-lactams has important therapeutic implications, as infections with such strains are now being more frequently treated with DAP-β-lactam combinations [34,35,36,38,42].

Several interesting themes emerged from our study. First, MprF mutations among DAP-R clinical strains have been noted in a variety of “hot spot” loci within this protein, most frequently (>50%) in its central bifunctional domain [28,30]. Of interest, recent in vitro studies [29] have confirmed that the presence of secondary *mprF* mutations can yield a decrease-of-function paradigm, featuring both reversal of DAP MICs, as well as a shift in PL profiles, paralleling DAP-S parental phenotypes. As noted above, prolonged LOX passage induces the accumulation of secondary mutations in *mprF*; however, the causal nature of this event for daptomycin resensitization was unclear, since other genetic mutations were also observed, most notably in *div1b* and *rpoC*. In this present study, we confirmed that these secondary *mprF* mutations (either via LOX passage or allelic exchange) are sufficient to restore parental level daptomycin MICs, as well as induce prototypical modifications in CM phenotypes. Studies are in progress to understand the mechanism(s) by which β-lactams can trigger the accumulation of secondary *mprF* mutations.

Second, DAP-R MRSA strains can modulate their surface charge toward a more relatively positive charge phenotype, potentially creating a “charge-repulsive” surface milieu against cationic molecules such as calcium-complexed DAP [26,27,28]. However, altered positive surface charge was only noted in two of our three strain-sets, and thus, it is not likely being determinative of the DAP-resensitization phenotype. Of note, β-lactams with PBP-1-targeting specificity may, in fact, not significantly alter surface charge, making their DAP-β-lactam synergy impacts independent of such events [38]. These data suggest that charge repulsion itself is not sufficient to fully explain the β-lactam resensitization of DAP-R strains.

Third, all three DAP-resensitized strains demonstrated substantially decreased CM fluidity as compared to their respective DAP-R strains and similar to their respective DAP-S parental strains. There are optimal biophysical metrics within the CM microenvironment that appear to maximize interactions of cationic peptides with the CM of MRSA [20,21,22,24]. Therefore, MRSA CMs containing extremes of rigidity/fluidity are comparatively resistant to interactions with such peptides [34,36,47]. The similar patterns of CM order of DAP-S parental and DAP-resensitized strains, on the one hand, vs. the distinctly different patterns of CM order in their respective DAP-R variants, underscored the likelihood that this phenotype is playing a relevant role in the overall differences in ultimate DAP-induced killing.

Finally, the above differences in CM order within the strain-sets were well correlated with changes in both carotenoid content and the patterns of anteiso-BCFAs; thus, changes in CM carotenoid content can lead to a buildup of C30 precursor species that shuttle into the menaquinone-fatty acid oxidation pathways [34]. In addition, CM–carotenoid interaction with the scaffold protein, flotillin, leads to CM microdomain formation, which is an important scaffolding for PBP maturation [48]; thus, the disassembly of these microdomains can have a major impact on MRSA antibiotic resistance [45]. Furthermore, it should be underscored that DAP inhibits the net synthesis of the cell envelope by interfering with such microdomains of DAP-S bacteria, leading to reorganization of the overall CM architecture, followed by the delocalization of essential CM proteins, such as the lipid II synthase, MurG [39,40]. Moreover, it is well known that various “stressors” can evoke a pattern of enhanced production of anteiso-BCFAs, with resultant increases in CM fluidity as a protective survival mechanism. Thus, Bacillus species can elicit such anteiso-BCFA shifts during ‘cold shock’ [49]. The shift we observed in anteiso-BCFAs and other FAs in the DAP-R mutants may represent the equivalent of a “cold shock” response at 37 °C. These same iso-to-anteiso-BCFA shifts, linked with perturbed CM order, have also been documented in previously studied *S. aureus* strains [35].

We recognize that the current investigation has several key limitations: (i) only three DAP S/DAP-R/DAP-resensitized strain-sets were studied; (ii) only a relatively focused cadre of phenotypic characteristics were interrogated in comparing the strain-sets; (iii) only a single β lactam antibiotic was used for prolonged passage, leaving unresolved whether other PBP-specific or PBP-promiscuous β-lactams can elicit the same adaptations; (iv) the linkage of our CM perturbations with specific metabolomics modifications was not explored [50]; and (vi) additional mutations documented previously in prolonged LOX-passaged strains (41) were not systematically investigated (e.g., via allelic exchange) to determine their impacts on the above CM parameters. It should be noted that DAP-resensitization did not occur post-LOX passage in DAP-R strains lacking a primary *mprF* mutation [41]; thus, it is highly likely that *mprF* and its associated CM changes play a critical role in the DAP-resensitization phenomenon.

## Figures and Tables

**Table 1:** List of study strains and their DAP MIC.

Strain Set ^a^	Strain Name	Strain Description	DAP MIC ^b^ (µg/mL)	LOX MIC ^c^ (µg/mL)	SNPs in *mprF* ^d^
I	C24	DAP-S	0.5	8	WT
	C25	DAP-R	2	4	S295L
	C25-LOX	DAP-resensitized (LOX passaged)	<0.125	8	S295L + L84 (Translocase domain)
	C25, *mprF* DM	Secondary *mprF* mutation (L84 ^e^) introduced into C25	0.125	16	S295L + L84 ^e^
II	D592	DAP-S	0.5	512	WT
	D712	DAP-R	2	512	L341S
	D712-LOX	DAP-resensitized (LOX passaged)	0.5	1024	L341S + S136L (Translocase domain)
	D712, *mprF* DM	Secondary *mprF* mutation (S136L) introduced into D712	0.5	1024	L341S + S136L
III	J01	DAP-S	0.5	16	WT
	J03	DAP-R	2–4	2	T345I
	J03-LOX	DAP-resensitized (LOX passaged)	0.125	32	T345I + R788L Synthase domain
	J03, *mprF* DM	Secondary *mprF* mutation (R788L) introduced into J03	0.125	16	T345I + R788L

^a^ Sets of isolates are represented by alternative shading and no shading, with the first strain in each set being the DAP-S parental strain, the second in each set being the DAP-R strain and the third and fourth being the DAP-resensitized strain generated by LOX passage or allelic exchange, respectively; ^b,c,d^ Data in this table have been previously published (41); ^e^ nonsense mutation (41).

**Table 2:** Phospholipid composition (%) of LOX passaged strain vs. DAP-R/DAP-S.

Strains	Total LPG	PG	CL
C24	12 ± 3	80 ± 6	8 ± 5
C25	25 ± 5 ^a^	70 ± 5 ^a^	6 ± 3
C25-LOX	5 ± 1 ^b^	94 ± 1 ^b^	2 ± 1 ^b^
C25, *mprF* DM	11 ± 2 ^c^	83 ± 4 ^c^	6 ± 6
D592	20 ± 3	77 ± 3	2 ± 3
D712	23 ± 2 ^a^	74 ± 4 ^a^	3 ± 2
D712-LOX	16 ± 4 ^b^	81 ± 5 ^b^	3 ± 2
D712, *mprF* DM	21 ± 2	75 ± 2	4 ± 1
J01	22 ± 2	70 ± 2	8 ± 2
J03	31 ± 7 ^a^	66 ± 6 ^a^	3 ± 1 ^a^
J03-LOX	20 ± 1 ^b^	78 ± 3 ^b^	3 ± 2 ^b^
J03, *mprF* DM	16 ± 3 ^c^	78 ± 4 ^c^	6 ± 1 ^c^

^a^*p*-value < 0.05; DAP-R vs. DAP-S; ^b^ *p*-value < 0.05; LOX passaged strains vs. DAP-R; ^c^ *p*-value < 0.05; *mprF* DM strains vs. DAP-R.

**Table 3:** Surface charge of study strains.

Strains	% Cytochrome C Unbound
C24	53 ± 1
C25	62 ± 0 ^a^
C25-LOX	54 ± 0 ^b^
C25, *mprF* DM	45 ± 0 ^c^
D592	56 ± 0
D712	85 ± 3 ^a^
D712-LOX	46 ± 1 ^b^
D712, *mprF* DM	57 ± 1
J01	58 ± 0
J03	48 ± 0 ^a^
J03-LOX	55 ± 0 ^b^
J03, *mprF* DM	44 ± 0 ^c^

^a^*p*-value < 0.05; DAP-R vs. DAP-S; ^b^ *p*-value < 0.05; LOX passaged strains vs. DAP-R; ^c^ *p*-value < 0.05; *mprF* DM strains vs. DAP-R.

**Table 4:** Fatty acids (%) composition of LOX passaged strain vs. DAP-R/DAP-S.

Strain Set ^a^	Iso FA	Anteiso FA	SFA
C24	30 ± 0.1	41 ± 0.12	25 ± 0.03
C25	27 ± 0.01 ^a^	44 ± 0.02 ^a^	22 ± 0.03 ^a^
C25-LOX	25 ± 0.07 ^b,c^	41 ± 0.1 ^b,c^	29 ± 0.3 ^b,c^
D592	24 ± 0.6	40 ± 0.03	31 ± 0.4
D712	25 ± 0.01 ^a^	40 ± 0.03	26 ± 0.2 ^a^
D712-LOX	25 ± 0.04 ^b^	45 ± 0.2 ^b,c^	31 ± 0.2 ^c^
J01	31 ± 0.03	40 ± 0.03	25 ± 0.02
J03	29 ± 0.1 ^a^	45 ± 0.2 ^a^	22 ± 0.11 ^a^
J03-LOX	27 ± 0.1 ^b,c^	43 ± 0.11 ^b,c^	26 ± 0.04 ^b,c^

^a^*p*-value < 0.05; DAP-R vs. DAP = S; ^b^ *p*-value < 0.05; LOX passaged strains vs. DAP-S; ^c^ *p*-value < 0.05; LOX passaged strains vs. DAP-R; SFA = Saturated/Straight Chain FAs.

**Table 5:** CM fluidity and carotenoid content of study strains.

Strains	CM Fluidity (PI Value)	Carotenoids (OD_450nm_)
C24	0.389 ± 0.01	0.685 ± 0.04
C25	0.370 ± 0.01 ^a^	0.261 ± 0.03 ^a^
C25-LOX	0.413 ± 0.00 ^b^	1.129 ± 0.24 ^b^
C25, *mprF* DM	0.368 ± 0.00	0.627 ± 0.02 ^c^
D592	0.408 ± 0.01	1.338 ± 0.01
D712	0.372 ± 0.01 ^a^	0.878 ± 0.01 ^a^
D712-LOX	0.389 ± 0.00 ^b^	1.037 ± 0.02 ^b^
C25, *mprF* DM	0.395 ± 0.00 ^c^	0.971 ± 0.03 ^c^
J01	0.381± 0.01	1.121 ± 0.04
J03	0.359 ± 0.01	0.697 ± 0.12 ^a^
J03-LOX	0.395 ± 0.01 ^b^	1.518 ± 0.27 ^b^
C25, *mprF* DM	0.430 ± 0.03 ^c^	1.545 ± 0.37 ^c^

^a^*p*-value < 0.05; DAP-R vs. DAP-S; ^b^ *p*-value < 0.05; LOX passaged strains vs. DAP-R; ^c^ *p*-value < 0.05; *mprF* DM strains vs. DAP-R.

## Supplementary Materials

The following are available online at https://www.mdpi.com/article/10.3390/microorganisms9051028/s1.
