# Supplementary material for: Cell Membrane Adaptations Mediate β-Lactam-Induced Resensitization of Daptomycin-Resistant (DAP-R) Staphylococcus aureus In Vitro
Source: Microorganisms. 2021 May 11;9(5):1028. doi: 10.3390/microorganisms9051028 (PMC8150363; doi:10.3390/microorganisms9051028)
Supplement: Supplementary file 1 [file microorganisms-09-01028-s001.zip › microorganisms-1213875-supplementary.pdf]

Supplemental Methods

Construction of mprF-mutants by allelic exchange

The introduction of a secondary *mprF* mutation into the three DAP-R backgrounds (which contain a single *mprF* mutation) was conducted using the allelic exchange protocol developed by Monk and Stinear [44] with modification (outlined below).

Oligonucleotides tailed with sequence complementary to pIMAY-Z were designed to amplify a ≈1.2kb region surrounding the secondary *mprF* mutation (table S2), the LOX passaged DAP-resensitized strains serving as a donor for the sequence. Amplicons were gel extracted and three separate Seamless Ligation Cloning Extracts (SLiCE) were performed into the linearized pIMAY-Z backbone [45]. Each 10 μL SLiCE reaction contained 1 μL of 10 × ligation buffer (NEB), 1 μL pIMAY-Z (50–100 ng/μL), 3 μL of gel extracted insert (20–30 ng/μL), 4 μL of dH2O, and 1 μL of SLiCE, incubated at 37 °C for 30 min.

The generation of competent cells (for *E. coli*): an overnight culture of *E. coli* strain IM08B [46] grown in Luria–Bertani broth (LB) was diluted 1:200 and incubated at 37 °C with agitation (200 rpm) to an optical density at 600 nm (OD_{600}) of 0.5–0.7. Culture were moved to ice (minimum 15 min) to arrest growth, with all subsequent steps performed at 4°C. Cells were collected by centrifugation (5,000 x g, 20 min), washed twice with an equal volume of autoclaved ice-cold dH_2O, then washed twice with an equal volume of ice-cold 10 % (v/v) glycerol in dH_20, and lastly resuspended 1:1000 in the original culture volume of ice-cold 10 % (v/v) glycerol in dH20. Aliquots of 40 μL were frozen at – 80 °C.

Before the transformation of IM08B, the SLiCE reaction was dialyzed for 10 min on a mixed cellulose ester membrane filter (0.025 μM, MF-Millipore). Electrocompetent IM08B cells were thawed on ice and 5 μL of SLiCE reaction was added to the cells; then, they were gently flicked to mix. Cells were transferred to a 1 mm electroporation cuvette (Bio-Rad) and electroporated at 1800 V, 200 Ω and 25 μF. Cells were immediately resuspended in 1 mL of LB broth and incubated at 37 °C for 1 hour. IM08B transformants were selected in LB supplemented with 10 μg/mL chloramphenicol, incubated overnight at 37 °C with agitation (200 rpm).

The FavorPrep Plasmid Extraction Mini Kit (Favorgen) was used to isolate pIMAY-Z, following the manufacturer's guidelines with the following exceptions: 25 mL of culture was run through each column, and 2.5 x volume of buffers FAPD1, FAPD2 and FAPD3 were used. Plasmids were concentrated using the Novagen Pellet Paint Co-Precipitant precipitation protocol.

To create the electrocompetent *S. aureus* cells, once the cells were diluted to an OD_{600} of 0.5 (Cell Density Meter, Biowave), they were incubated for ≈1.5 hours to an OD_{600} of ≈1.0. Concentrated pIMAY-Z was electroporated into the respective DAP-R strains (J03, D712 and C25) according to the published protocol [44].

Allelic exchange was performed following the "Slow (2015) Integration" approach [44]. At the final stage, white colonies (potential *mprF*-double mutants (DM)) were screened on MH agar supplemented with 4 μg/mL daptomycin in parallel grown on BHI agar.

Suspected *mprF* DM colonies and cultures of the parent DAP-R strains used for allelic exchange underwent whole-genome sequencing

(WGS) to confirm their genotype, performed as previously described [41]. Briefly, gDNA was extracted from a single colony (Chemagic DNA/RNA kit, Perkin Elmer), WGS libraries prepared (Nextera XT DNA preparation kit, Illumina), and sequencing conducted on a NextSeq (Illumina) with 2 x 150 bp chemistry. The short-read sequence data were mapped to complete reference genomes J01 (CC8, RefSeq accession NZ_CP040619.1) or D592 (CC5, NZ_CP040665.1), and mutations were identified using Snippy v4.6.0 (https://github.com/tseemann/snippy). Both the primary (from the DAP-R parent) and secondary (introduced) *mprF* mutations were confirmed in all three backgrounds. Only one off-target missense mutations was identified: a A308V amino acid change in predicted gene FFX42_RS09315 in the D712 *mprF* DM (reference D592, CC5 background).

Table S1. Oligonucleotides used in allelic exchange protocol.

Oligonucleotide name	5' to 3' sequence
pIMAY-Z_C25_F	CCTCACTAAAGGGAACAAAAGCTGGGTACCTGGTGCTTATAATGTTGGGC
pIMAY-Z_C25_R	CGACTCACTATAGGGCGAATTGGAGCTCGACGTAACATCTTTAGCAGG
pIMAY-Z-J03_F	CCTCACTAAAGGGAACAAAAGCTGGGTACCCGTTAGGTGATGAAAATGCC
pIMAY-Z-J03_R	CGACTCACTATAGGGCGAATTGGAGCTCGACCCATTAGATACAGGTGG
PIMAY-Z-D712_F	CCTCACTAAAGGGAACAAAAGCTGGGTACCAAGAAGCACTCATAATCGGC
PIMAY-Z-D712_R	CGACTCACTATAGGGCGAATTGGAGCTCCGTAAGTGAAGAATGTCGCC

Table S2. Fatty acid (FA) composition (%) of LOX-passaged strain.

FAs	C24	C25	C25-LOX	D592	D712	D712-LOX	J01	J03	J03-LOX
**% of Iso FAs**									
**14:0**	9 ± 0.0	7 ± 0.01[a]	7 ± 0.04[b,c]	5 ± 0.2	5 ± 0.0[a]	7 ± 0.03[b,c]	11 ± 0.03	8 ± 0.1[b]	8 ± 0.04[b,c]
**15:0**	15 ± 0.02	14 ± 0.01[a]	12 ± 0.01[b,c]	14 ± 0.02	15 ± 0.02[a]	14 ± 0.01[c]	13 ± 0.02	13 ± 0.1[b]	13 ± 0.1[c]
**16:0**	3 ± 0.01	3 ± 0.0[a]	2 ± 0.01[b,c]	2 ± 0.02	2 ± 0.0	1 ± 0.01[b,c]	4 ± 0.0	4 ± 0.0[b]	3 ± 0.0 [b,c]
**17:0**	3 ± 0.01	4 ± 0.0[a]	3 ± 0.01[c]	2 ± 0.01	4 ± 0.01[a]	2 ± 0.0[b,c]	3 ± 0.01	4 ± 0.0[b]	4 ± 0.0 [b,c]
**Total iso FA**	30 ± 0.1	27 ± 0.01[a]	25 ± 0.07[b,c]	24 ± 0.6	25 ± 0.01[a]	25 ± 0.04[b]	31 ± 0.03	29 ± 0.1[b]	27 ± 0.1[b,c]
**% of Anteiso FAs**									
**15:0**	4 ± 0.0	5 ± 0.01[a]	5 ± 0.01[c]	38 ± 0.1	40 ± 0.1[a]	37 ± 0.03 [b,c]	36 ± 0.0	39 ± 0.2[a]	37 ± 0.11 [b,c]
**17:0**	37 ± 0.08	39 ± 0.03[a]	37 ± 0.2 [b,c]	4 ± 0.0	5 ± 0.01[a]	3 ± 0.0 [b,c]	4 ± 0.0	5 ± 0.01[a]	5 ± 0.0[b]
**Total Anteiso FA**	41 ± 0.12	44 ± 0.02[a]	42 ± 0.2 [b,c]	41 ± 0.1	45 ± 0.1[a]	40 ± 0.03 [b,c]	40 ± 0.03	45 ± 0.2[a]	43 ± 0.11 [b,c]
**% of SFAs**									
**14:0**	1 ± 0.01	1 ± 0.0[a]	1 ± 0.0 [b,c]	1 ± 0.1	1 ± 0.01[a]	2 ± 0.0 [b,c]	1 ± 0.01	1 ± 0.01	1 ± 0.01
**16:0**	4 ± 0.0	3 ± 0.0[a]	4 ± 0.01 [b,c]	4 ± 0.0	4 ± 0.0[a]	7 ± 0.01 [b,c]	5 ± 0.01	4 ± 0.02[a]	4 ± 0.02 [b]
**17:0**	1 ± 0.0	1 ± 0.01[a]	1 ± 0.01 [b,c]	1 ± 0.0	1 ± 0.1[a]	2 ± 0.0 [b,c]	1 ± 0.01	1 ± 0.0	2 ± 0.0 [b,c]
**18:0**	11 ± 0.02	10 ± 0.01[a]	13 ± 0.09[b,c]	12 ± 0.0	12 ± 0.0[a]	13 ± 0.02 [b,c]	12 ± 0.02	12 ± 0.1[a]	12 ± 0.1[b]
**19:0**	2 ± 0.01	2 ± 0.01	3 ± 0.05 [b,c]	2 ± 0.02	2 ± 0.02[a]	3 ± 0.01 [b,c]	2 ± 0.0	1 ± 0.02[a]	3 ± 0.02 [b,c]
**20:0**	6 ± 0.02	6 ± 0.03	7 ± 0.2[b]	5 ± 0.04	5 ± 0.04[a]	5 ± 0.02 [b,c]	4 ± 0.01	4 ± 0.04[a]	4 ± 0.04*
**Total SFA**	25 ± 0.03	22 ± 0.03[a]	29 ± 0.3 [b,c]	31± 0.4	26 ± 0.2[a]	31 ± 0.2[c]	25 ± 0.02	22 ± 0.11[a]	26 ± 0.04 [b,c]

[a] P-value < 0.05; DAP-R vs. DAP=S;. [b] P-value < 0.05; LOX passaged strains vs. DAP-S. [c] P-value < 0.05; LOX passaged strains vs. DAP-R. SFA=Saturated/Straight Cha
